# 

**Published:** 1855-05

**Authors:** 


### IOWA
### MEDICAL JOURNAL.

CONDUCTED BY THE FACULTY OF THE

MEDICAL DEPARTMENT OF THE IOWA UNIVERSITY.

PUBLISHED EVERY ALTERNATE MONTH.

TERMS—TWO DOLLARS, IN ADVANCE.

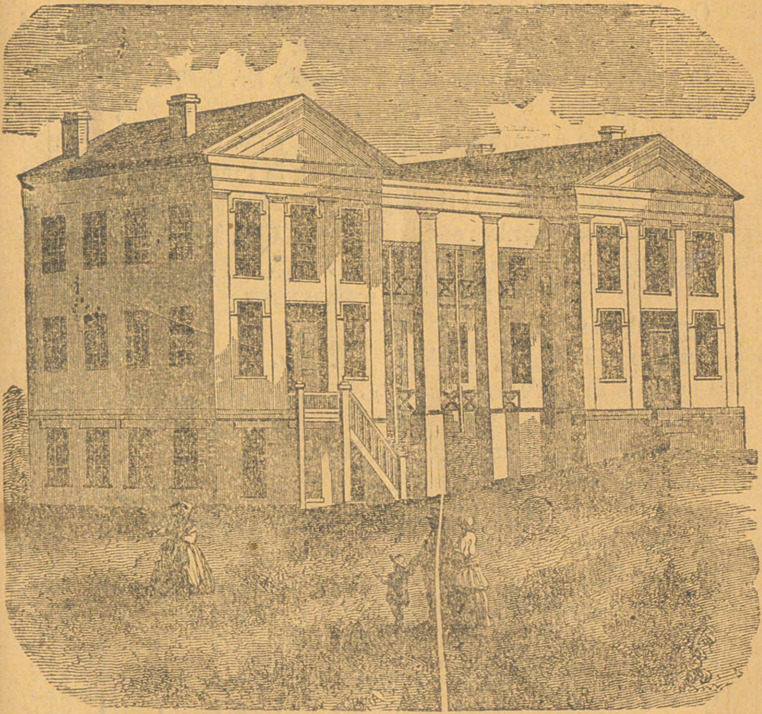

KEOKUK, IOWA:

PRINTED AT THE WHIG BOOK AND JOB OFFICE.

Address all Communications, post-paid, to "Medical College, Box 109, Keokuk, Iowa.

